# Anti-CCP Antibodies Are Not Associated with Familial Mediterranean Fever in Childhood

**DOI:** 10.1155/2013/498581

**Published:** 2013-09-09

**Authors:** Hatice Onur, Hale Aral, Vefik Arica, Gamze Bercem, Murat Usta, Özgur Kasapcopur

**Affiliations:** ^1^Department of Pediatric, Istanbul Training and Research Hospital, Istanbul, Turkey; ^2^Department of Biochemistry, Istanbul Training and Research Hospital, 34080 Istanbul, Turkey; ^3^Department of Pediatric, Mustafa Kemal University Medical Faculty, 31000 Antakya, Turkey; ^4^Department of Biochemistry, Giresun University Medical Faculty, 28000 Giresun, Turkey; ^5^Department of Pediatric Rheumatology, Istanbul University Cerrahpasa Medical Faculty, Istanbul, Turkey

## Abstract

*Objective*. Anticyclic citrullinated peptide antibodies (anti-CCP) testing is useful in the diagnosis of rheumatoid arthritis (RA) with high specificity. Arthritis is a very common clinical manifestation in children with familial Mediterranean fever (FMF). The aim of the study was to show the presence of anti-CCP antibodies in child individuals diagnosed with FMF. *Material and Methods*. The study groups comprised one hundred and twenty-six patients (126) diagnosed with FMF (female/male (*n*): 66/60) and 50 healthy controls (female/male (*n*): 25/25). Clinical and laboratory assessments of the FMF patients were performed during attack-free periods. Erythrocyte sedimentation rate (ESR), serum C-reactive protein (CRP), fibrinogen, and anti-CCP antibody levels were measured. *Results*. Anti-CCP was negative in healthy controls and also in all FMF patients. There was not a significant difference in anti-CCP between the patient and the control groups. Our study has shown that anti-CCP was correlated moderately with age (rs = 0.271; *P* = 0.0020), duration of illness (rs = 0.331; *P* < 0.0001), and colchicine therapy (rs = 0.259; *P* = 0.004). *Conclusion*. Our data show that anti-CCP antibodies are not associated with FMF. Anti-CCP does not have a priority for identifying FMF arthritis from the other inflammatory arthritis.

## 1. Introduction

 Familial Mediterranean fever (FMF) is an autosomal recessive disease that is prevalent among eastern Mediterranean populations, mainly non-Ashkenazi Jews, Armenians, Turks, and Arabs [1]. Patients suffer from recurrent self-limited inflammatory febrile attacks, abdominal, chest, or joint pain. It is still unknown what triggers or ends these periodical attacks [2, 3].

 Cyclic citrullinated peptide (CCP) autoantibodies bind antigenic determinants that contain unusual amino acid citrulline. Citrulline is a nonstandard amino acid as it is not incorporated into proteins during protein synthesis [4]. Citrullination or deimination is an enzyme-catalysed process in which the positively charged NH_2_-group of amino acid arginine is hydrolyzed to a neutral oxygen group. It is this oxygen group of peptidylcitrulline that is specifically recognized by autoantibodies in rheumatoid arthritis (RA). The citrulline residues are essential part of the antigenic determinants recognized by the RA antibodies. So anti-CCP testing is particularly useful in the diagnosis of RA with high specificity present early in the disease process [5, 6].

 Although anti-cyclic citrullinated peptide (anti-CCP) levels were investigated in several times in rheumatoid arthritis, rare investigation in FMF arthritis has been done. Arthritis seen in FMF patients is generally acute monoarthritis which is predominantly affecting the lower limbs, and it occurs during attack periods. Generally this arthritis is without sequela, but a few patients develop chronic, destructive arthritis [7, 8].

 The aim of the study was to show the presence of anti-cyclic citrullinated peptide (anti-CCP) antibodies in children, diagnosed with Familial Mediterranean Fever (FMF).

## 2. Material and Methods

 The study group included 126 patients with diagnosis of FMF attended to Istanbul Cerrahpasa Medical Faculty, Pediatric Rheumatology Clinics. Patients with other known chronic diseases were excluded. None of the patients were in attack period of FMF. The control group comprised 50 ages and sex-matched healthy children. The blood samples from healthy controls were taken for another reason with parent approval. The diagnosis of FMF was established according to criteria defined by Yalçinkaya et al. [9]. The study was approved by the Local Ethics Committee of Istanbul Education and Research Hospital and was in accordance with the Helsinki Declaration of 1983. Blood samples from patients with FMF and the control group were drawn. In Istanbul Education and Research Hospital Central Biochemistry Laboratory, anti-CCP IgG levels were measured by enzyme-linked immunoassay (Euroimmun Mediziische Labordiagnostika AG, Lübeck, Germany). Including dilution before analysis (1 : 101 with sample buffer), patient samples were assayed automatically, and absorbances were taken in 450 and 620 nm. Relative concentrations of serum anti-CCP levels as “RU/mL” were found with calibration at 5 points. Lower detection limit was 0.3 RU/mL. According to the kit insert, intraassay coefficient values (CV) were as follows: 5.9% at a mean level of 18 RU/mL and 3.4% at a mean level of 52 RU/mL. And interassay CV values were as follows: 6.3% at a mean level of 19 RU/mL and 6.8% at a mean level of 63 RU/mL. The cut-off value of 5 relative units was established. Routine biochemical analyses of serum and hour samples were done using Advia 2400 chemistry system; fibrinogen was determined using Sysmex Ca 1500 automatic coagulometer, and C-reactive protein (CRP) was measured by BNII nephelometer (Siemens Healthcare Diagnostics Inc, USA). ESR was measured automatically by Kimased Auto 60-Vacutest Kima system.

## 3. Statistics

 Statistical analysis was done with Number Cruncher Statistical System 2004 (NCSS systems, Kaysville, UT, USA) and MedCalc (MedCalc software, Broekstraat, Mariakerke, Belgium). Kolmogorov-Smirnov goodness-of-fit test was used to determine whether distributions of the variables were Gaussian. All the numerical variables were expressed as a mean ± SD if normally distributed or else as a median (25th and 75th percentiles). Pearson's chi-square test was used to compare categorical variables. Statistical comparisons of two groups were performed with Student's *t*-test for data with a Gaussian distribution or Mann-Whitney *U*-test for data with a non-Gaussian distribution. Pearson's correlation coefficient (*r*) or Spearman's rank correlation coefficient (rs) or biserial correlation coefficient (rb) was used to evaluate the degree of association between two variables. Two-way ANOVA was performed to identify if there is a significant interaction effect between the independent variables. All statistical tests were two-sided, and *P* values less than 0.05 were considered to indicate significance.

## 4. Results

 Patients and the control group were similar in age and sex, respectively, 11 (range 8–14) years 66 female/60 male and 11 (range 6–14) years 25 female/25 male. In the FMF patients, time for diagnosis of FMF was 6 (range 4–10) years, and duration of complaints was 5 (range 3–8) years. Of 126 patients, 56 had joint involvement and none of them had renal involvement.

 The presenting symptom was abdominal pain/fever for 51 patients, abdominal pain/fever/arthritis/arthralgia for 36, arthritis/arthralgia for 10, abdominal pain/fever/skin lesions for 6, fever/arthritis/arthralgia for 6, and the other symptoms (chest pain and myalgia/skin lesions) for 17 patients. 57(45%) patients have family history for FMF. Clinical and demographic characteristics and laboratory parameters of patients are shown in (Table 1).

 The anti-CCP levels in patient group and control group were 0.97 (0.70–1.39) RU/mL and 1.22 (0.79–1.5) RU/mL respectively (*P* = 0.125). The anti-CCP levels were not a significantly different in male and female subgroups in patient and control groups (*P* = 0.107 and *P* = 0.193, resp., Table 1).

 26 patients were homozygous for M694V, 38 were heterozygous for M694V, 46 have the other genetic mutations, and 16 patients were negative for any genetic mutations.

 We detected that anti-CCP was correlated moderately with age (rs = 0.271; *P* = 0.0020), and duration of illness (rs = 0.331; *P* < 0.0001), colchicine therapy (rs = 0.259; *P* = 0.004). Also poor positive correlations between fibrinogen and anti-CCP levels were detected (rs = 0.192; *P* = 0.0330).

 Anti-CCP values were negative in all FMF patients as well as in all healthy controls. 

 We detected anti-CCP values for FMF patient with arthritis (*n* = 62) and without arthritis (*n* = 64) to be 1.11 (0.72–1.48), 0.92 (0.65–1.19) RU/mL, respectively. The values less than 20 RU/mL accepted as negative. Anti-CCP values were not significant between FMF patients with or without arthritis (*P* = 0.148).

## 5. Discussion

 In recent years, several studies were established showing the superiority of anti-CCP in rheumatoid arthritis (RA) and other inflammatory diseases. As FMF is a low grade inflamation having articular involvement, we aimed to establish the association between FMF and anti-CCP values.

 There are studies in the literature investigating the value of anti-CCP in adult FMF patients [10–13]. All of these studies include adult patient groups and originate from our country where FMF is more frequent as being a Mediterranean country. Our study is the first data that is establishing the anti-CCP levels in children diagnosed with FMF.

 In our study, Anti-CCP was negative in healthy controls and also in all FMF patients. There was not a significant difference in anti-CCP between the patient and the control groups. In a widespread study including FMF patients, Tunca et al. have indicated that patients with the M694V/M694V genotype were found to have an earlier age of onset and higher frequencies of arthritis and arthralgia compared with the other groups (both *P* < 0.001) [1]. In our study no significant difference was detected between four mutation groups and anti-CCP levels (*P* : 0.849). Similar to our study Guler et al. suggested that autoantibody positivity is similar to the healthy population in FMF and it is thought that although MEFV mutations affect clinical course in other autoantibody mediated diseases, it is not related to autoantibody formation in FMF. Also Karatay et al. suggested that anti-CCP antibodies are not associated with FMF [10, 11].

 Ceri et al. showed anti-CCP prevalence is higher in FMF patients with arthritis than without arthritis and that a significant proportion of FMF patients with arthritis (13.5%) had moderate-high titers of anti-CCP. They conclude that anti-CCP antibodies may not be a reliable indicator to differentiate between FMF arthritis and rheumatoid arthritis [12]. In our study there was not significance between patients with or without arthritis and anti-CCP levels (*P* = 0.148). Uyanik et al. suggested that it is possible that long-term followup of the FMF patients with anti-CCP antibodies may reveal the eventual development of inflammatory joint disease. They established significant difference in anti-CCP between the patient and the control groups (*P* = 0.008) and positive correlation between arthritis and anti-CCP (*P* = 0.001) [13].

 Arthritis seen in FMF patients is generally acute monoarthritis which predominantly affects the lower limbs, and it occurs during attack periods. Generally this arthritis is without sequela, but few patients develop chronic, destructive arthritis [7, 8]. Anti-CCP is detected as the best diagnostic antibody in the diagnosis of RA and was predictive in estimating the persistence of the disease and the probability of radiological destruction in the early arthritis [14]. Other studies reported the sensitivity of anti-CCP in RA as 41–67% and specificity as 90–98% [15–18].

 In a previous study established in the pediatric age group, Kasapçopur et al. indicate that anti-CCP positivity is found in RF positive patients with erosive joint disease. This study suggested that anti-CCP determination should not be used as a screening method for patients with juvenile idiopathic arthritis, but it can be used to predict erosive joint damage, especially in children with polyarticular disease [19]. In our study we detected anti-CCP values for FMF patient with arthritis (*n* = 62) and without arthritis (*n* = 64) to be 1.11 (0.72–1.48), 0.92 (0.65–1.19) RU/mL, respectively. Anti-CCP values were not significant between FMF patients with or without arthritis (*P* = 0.148). As a result of this finding we suggest that anti-CCP levels are not descriptive to distinguish FMF arthritis from the other arthritis types in children.

 The valance of anti-CCP, especially in polyarticular JIA has been highlighted in many studies [20–22]. Gupta et al. indicate that anti-CCP antibodies can be used as a marker to predict severe course of JIA at the onset to guide optimal aggressive therapy [23].

 Lately not only the patients but also the relatives of FMF patients were investigated for anti-CCP. Kim et al. detected anti-cyclic citrullinated peptide (anti-CCP) antibody in unaffected first degree relatives (FDRs) of rheumatoid arthritis (RA) patients and showed that the levels of anti-CCP were significantly higher than those of the healthy controls [24].

 Our study has shown that anti-CCP was correlated moderately with age (rs = 0.271; *P* = 0.0020), duration of illness (rs = 0.331; *P* < 0.0001), and colchicine therapy (rs = 0.259; *P* = 0.004). The average age for FMF patients attended to our study was 11 years (min 6–max 14), and we detected positive correlation between age and anti-CCP values. It is thought that this positive correlation calls for more accurate investigations that should be done in elder ages of FMF patients. Low anti-CCP values should be associated with low autoantibody titers seen in young ages. Also poor positive correlation between fibrinogen and Anti-CCP levels was detected (*P* : 0.0330  *r* : 0.192).

 In conclusion, no published data in children establish anti-CCP values in patients with FMF compared with healthy controls. Our data show that anti-CCP antibodies are not associated with FMF and does not have a priority for identifying FMF arthritis from the other inflammatory arthritis in childhood.

## Figures and Tables

**Table 1:** Demographic characteristics and laboratory parameters, anti-CCP levels according to gender.

	Patient group (*n* = 126) mean ± SD median (IQR)	Control group (*n* = 50) mean ± SD median (IQR)
Age (years)	11 ± 4	10 ± 4
Female/male (*n*)	66/60	25/25
Duration of diagnosis (years)	7 ± 4	
Duration of complaints (years)	6 ± 3	
Medication* (years)	4 ± 2	
Fibrinogen (mg/dL) (150–400)	308 ± 87	
CRP (mg/dL) (0–4.99)	3.0 ± 12.8	
ESR (mm/hour)	20 ± 15	
Genetic test (*n*):		
M694V/M694V	26	
M694V/other	38	
Other/other	46	
Negative	16	
Anti-CCP(RU/mL) Female/male	0.92 (0.64–1.33)/1.10 (0.73–1.48)	1.27 (1.07–1.51)/1.07 (0.68–1.49)
* P *	0.107	0.193

*Colchicine treatment.
